# Binding studies of creatinine and urea on iron-nanoparticle

**DOI:** 10.1186/s40064-015-1452-2

**Published:** 2015-11-19

**Authors:** Biswadip Banerji, Sumit Kumar Pramanik

**Affiliations:** Organic & Medicinal Chemistry Division, CSIR-Indian Institute of Chemical Biology, 4 Raja S. C. Mullick Road, Jadavpur, Kolkata, 700032 India; Academy of Scientific and Innovative Research (AcSIR), CSIR-Indian Institute of Chemical Biology Campus, 4 Raja S. C. Mullick Road, Kolkata, 700032 India

## Abstract

Kidney diseases are complicated and can be fatal. Dialysis and transplantation are the only survival solutions to the patients suffering from kidney failures. Both hemodialysis and peritoneal dialysis are risky, due to the possibility of infection and these are expensive and time consuming. The development of simple and reliable technique for the clearance of creatinine and urea from the body is an important part of biotechnology. We have synthesized an iron nanoparticle (INP) and studied its binding with creatinine and urea. The DLS, TEM, AFM, FT-IR and Powder-XRD studies demonstrate strong binding of creatinine and urea to the nanoparticles. This finding may be helpful if it is used in the dialysis technologies. The proposed method may substantially decrease dialysis time and improve its quality in terms of urea and creatinine clearances.

## Background

Chronic kidney disease (CKD) is becoming increasingly common and can lead to chronic kidney failure when both the kidneys stop functioning totally (Dousdampanis et al. 2014; Rahbari-Oskoui et al. 2014). The kidneys are filtration units which purify blood from the waste by-products including creatinine and urea. Eventually in the case of kidney failure these and other waste molecules accumulate in the body and dialysis becomes the only solution (Kim and Takayama 2015; Nassar and Ayus 2001). To date no proper medicine has emerged for the treatment of CKD, with the exception of strong immuno-suppressant drugs which actually prolong the kidney failure rather than effect a cure (Nassar and Ayus 2001; Dalal et al. 2011). Dialysis is associated with a high risk of infection and is costly (Nassar and Ayus 2001).

Among various nanoparticles, iron nanoparticles (INPs) have been found to be biologically compatible, and can be coated with biomolecules, drugs, enzymes, or antibodies to increase their functionalities for bio-sensing applications (Xie et al. 2010; Todd et al. 2014; Bachelet-Violette et al. 2014; Verma et al. 2014). It is cheap, non-toxic, and easy to prepare (Tromsdorf et al. 2009; Banerji et al. 2012a, b). Now a days INPs have been widely used for targeted drug delivery, immobilization of enzymes, biosensor, and different environmental analysis (Verma et al. 2014). They are extensively used as MRI contrast agents due to their ability to shorten T_2_* relaxation times for the liver, spleen, and bone marrow (Na et al. 2009). The surface properties of INPs allow to functionalize with small organic molecules by their various functional groups for wide range of applications (Weingart et al. 2013). The nano-bio composite of INPs and chitosan have been reported for the detection of glucose, urea, phenolic compounds and ferritin (Lin et al. 2015). However, immobilization of biomolecules onto surface charged super paramagnetic INPs are of great interest because of magnetic behavior of their bio conjugates (Wahajuddin 2012).

Recently, we have initiated a program to synthesize iron-nanoparticles (INPs) with high affinity towards nitrogen containing compounds (Banerji et al. 2012a). With an aim to test the use of the INPs for the treatment of CKD patients, we have studied the binding affinity of creatinine and urea using various techniques like DLS, TEM, AFM, FT-IR and Powder-XRD. Here we disclose our preliminary results on the binding studies of creatinine and urea on INPs. One future possibility of this study is to utilize the strong affinity binding of creatinine and urea into the dialysis technology. It is proposed that in the presence of nanoparticles the rate of osmosis may be much higher compared to normal case leading to better dialysis. The present study thus may be the first foot-step towards achieving that major goal.

## Methods

### Materials

FeCl_2_·4H_2_O, FeCl_3_·6H_2_O, urea and creatinine were purchased from Sigma Aldrich. Sodium hydroxide (NaOH), hydrochloric acid (HCl), citric acid and sodium citrate were obtained from Merck. Carbon coated copper grid for TEM study was purchased from Allied Scientific Product, USA. ASTM V1 Grade Ruby Mica sheet for AFM study was purchased from Micafab India Pvt. Ltd., Chennai, India. Water obtained from Sartorius Stedim biotech machine was used for all the experiments.

### Synthesis of iron-nanoparticles

First 1 g (0.005 mol) FeCl_2_·4H_2_O was dissolved in 20 ml 1 (M) HCl and then 2.7 g (0.01 mol) FeCl_3_.6H_2_O was added to the solution and stirred under magnetic stirrer. Then 2 (M) NaOH solution was added until the black precipitate appeared and the pH also become basic. This black precipitate was then separated and washed three times with 30 ml deionized and deoxygenated water. After that 20 ml citric acid-sodium citrate buffer was added to disperse the INPs. These INPs were washed by repeated cycles (four times) of centrifugation and dried in air. Millipore water was used throughout to disperse the nanoparticles as needed for characterization as well as further studies.

### Synthesis of iron-nanoparticle-creatinine and iron-nanoparticle-urea conjugates (Chen and Kimura 1999; Brust et al. 1994)

Creatinine and urea bind to the INPs by noncovalent interaction (van der Waals forces) between the INPs and creatinine/urea. Creatinine/urea solution of concentration 0.5 mg/ml was prepared by dissolving 50 mg of creatinine/urea in 100 ml millipore water. For the conjugation of creatinine/urea onto iron nanoparticles (INPs), 10 ml of this solution and 20 mg of as-synthesized INPs were taken into a flat-bottomed conical flask with glass stopper. The flask was sealed with parafilm and stirred at 60 °C for 24 h. The unbound creatinine and urea were separated by centrifugation at 30,000 rpm for 30 min. The conjugated INPs were washed by repeated cycles of centrifugation and dried under vacuum.

### Dynamic light scattering (DLS)-based zeta-potential measurements (Kuypers et al. 2015)

To obtain an idea about the size distributions and stability of INPs, creatinine conjugated INPs and urea conjugated INPs, DLS experiments (model: Zetasizer Nano Z, Malvern Instruments Ltd, United Kingdom) were carried out with their aqueous suspension at 20 °C. The scattered lights were collected at a 90^o^ angle. Data were acquired and analyzed by Precision Deconvolve program. For a typical DLS experiment, 200 μl (conc. 2 mg/ml) of a sample solution was slowly pipetted into a clean quartz micro-cuvette.

### TEM sample preparation and imaging (Banerji et al. 2014)

A Tecnai G2 Spirit TEM operating at 80 kV was used to study the morphology of the INPs and conjugated INPs. 10 µl (conc. 2 mg/ml) of the nanoparticle solution was placed on a 300-mesh carbon coated copper grid (formvar foil upon copper grids) and the excess samples were removed cautiously by tissue paper. No additional staining was done.

### AFM sample preparation and imaging (Banerji et al. 2012a)

10 µl (conc. 2 mg/ml) of the nanoparticle solution was deposited on a freshly cleaved muscovite Ruby mica sheet 10 min after that the sample was dried under vacuum. AC-mode atomic force microscopy was performed by using a Pico Plus 5500 AFM (Agilent Technologies, Inc., Santa Clara, CA, USA) with a piezo scanner maximum range of 9 μm. Microfabricated silicon cantilevers of 225 μm in length with a nominal spring force constant of 21–98 N/m were used from nanosensors. Cantilever oscillation frequency was tuned into resonance frequency. The cantilever resonance frequency was 150–300 kHz. All the images (512 × 512 pixels) were captured with a scan size between 0.5 and 5 μm at the scan speed rate of 0.5 rpm. The images were processed by flattening using Pico view software (Molecular Imaging Inc., Ann Arbor, MI, USA).

### FT-IR experiment (Banerji et al. 2013)

The FT-IR spectra of the samples were recorded on a JASCO FT/IR 4200 spectrometer using the (KBr) disc technique. For FT-IR measurements all the samples are used in their solid powder form. The solid creatinine, urea, creatinine conjugated INPs and urea conjugated INPs were mixed with KBr separately in a clean glass mortar and compressed to obtain a pellet. Background spectra were obtained with a KBr pellet for each sample. JASCO software was used for data processing.

### Powder X-Ray diffraction study

X-Ray diffraction studies of INPs, creatinine tagged INPs and urea tagged INPs were carried out with a Scifert X-ray diffractometer (C 3000) using ‘Cu ka’ radiation. The data collection was recorded in the range of 2θ = 5–60° with a step of 0.02^o^ and 2 s/step.

## Results and discussion

### DLS study

The average diameters and the size distribution polydispersity index (PDI) of the samples were determined by DLS. DLS experiments were carried out with their aqueous solutions and the results showed that the mean particle size of INPs (intensity average) are 8.66 ± 4 nm with PDI 0.23, creatinine tagged INPs are 9.26 ± 3 nm with PDI 0.29 and that of urea tagged INPs are 9.16 ± 4 nm with PDI 0.34. The zeta potential distribution of INPs are negative charged to −38.8 mV, creatinine tagged INPs with a negative charge −23.5 mV and of urea tagged INPs with a negative charge −21.4 mV in water, which are sufficient to keep the particles from interacting with each other and therefore maintain a stable particle size of the sample. The decrease in zeta potential of INPs is observed due to the coating of creatinine/urea over INPs which minimizes the free surface energy by restructuring the surface (Nel et al. 2009). The resulting negative charges in INPs, creatinine tagged INPs and urea tagged INPs are attributed to negative surface charge on them.

### TEM and AFM imaging

**Fig. 1:**
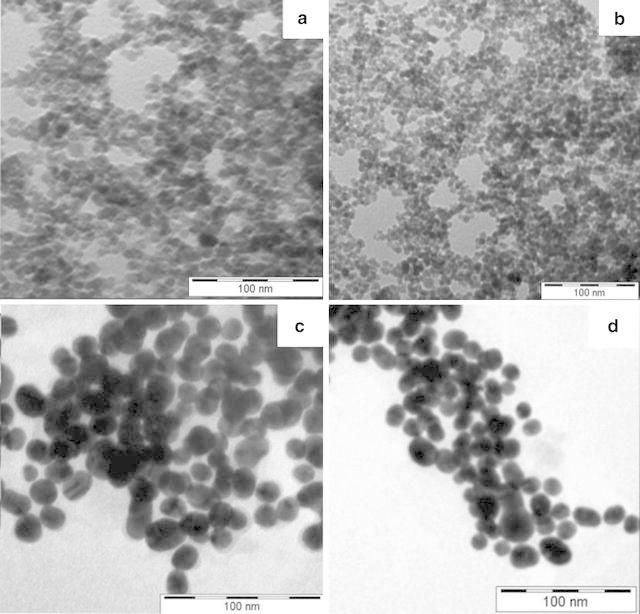
**a**, **b** TEM images of INPs; **c** TEM images of creatinine conjugated INPs and **d** TEM images of urea conjugated INPs

**Fig. 2:**
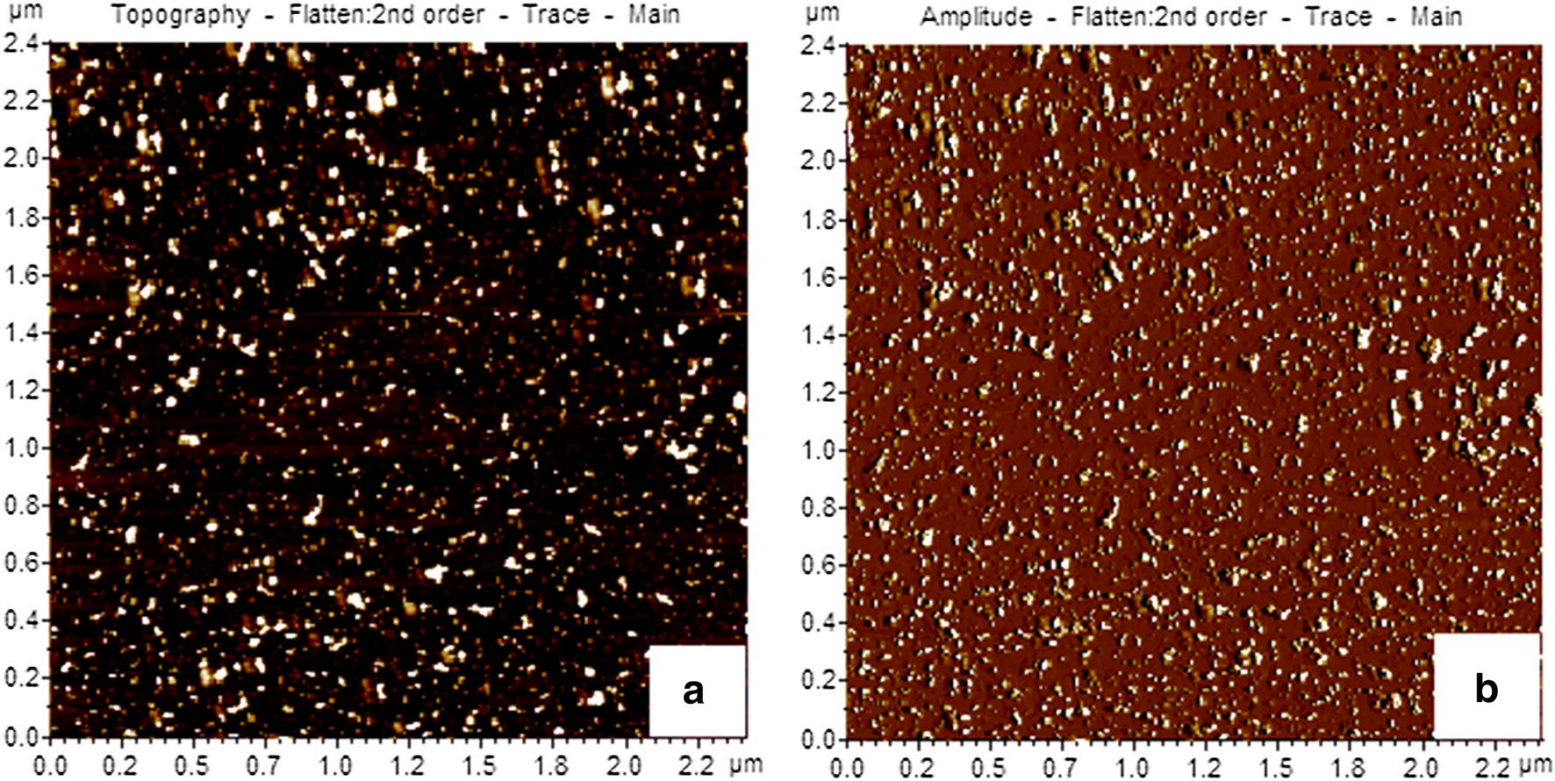
**a**, **b** AFM images of INPs

**Fig. 3:**
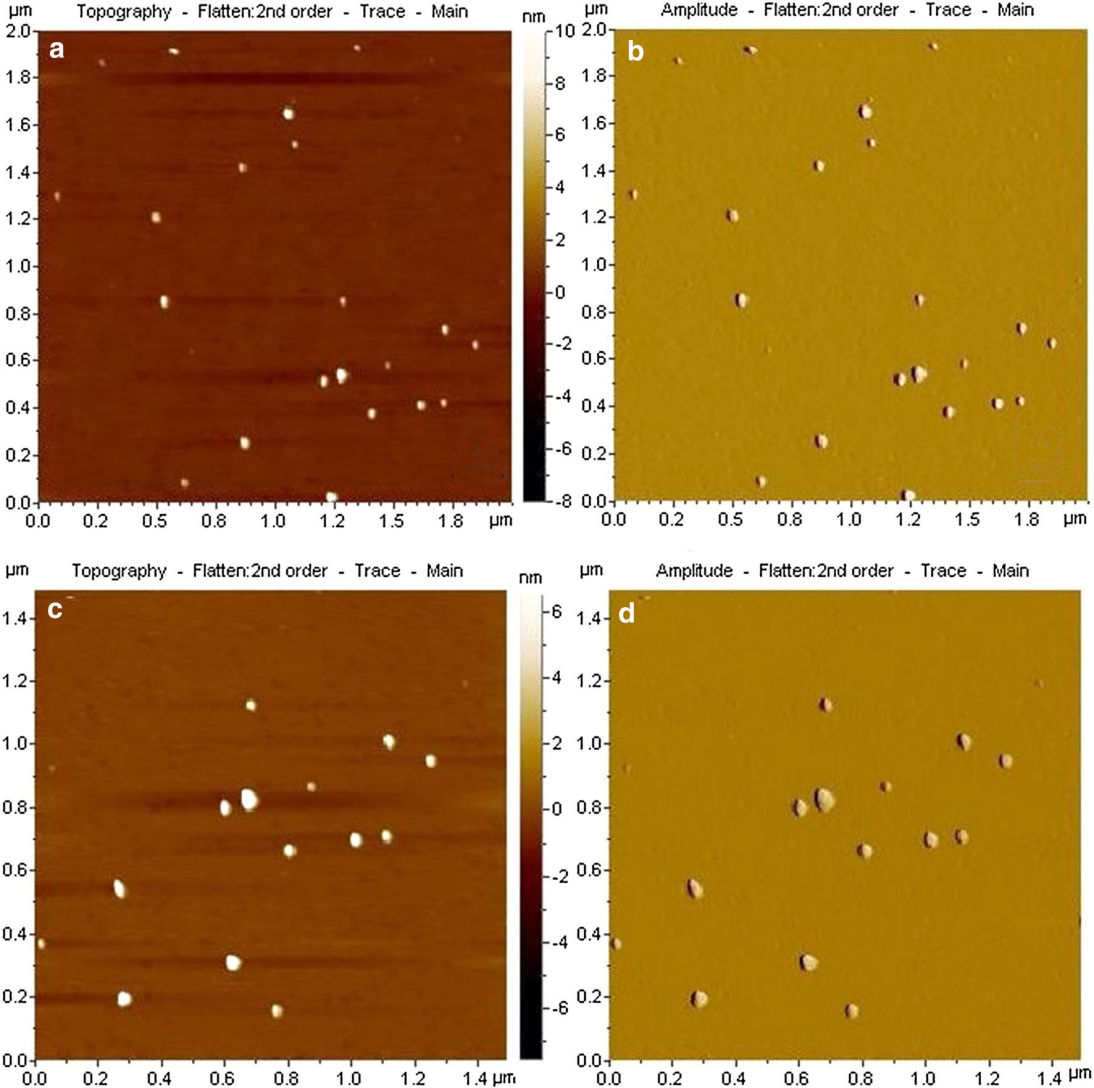
**a**, **b** AFM images of creatinine conjugated INPs and **c**, **d** AFM images of urea conjugated INPs

The transmission electron microscopy (TEM) images of INPs (Fig. 1a, b), creatinine conjugated INPs (Fig. 1c) and urea conjugated INPs (Fig. 1d) are depicted. It is observed from the figure that the size of INPs is almost uniform in nature and most of them are approximately spherical in size with diameter 6 nm. Figure 1 c, d show the size of creatinine conjugated INPs and urea conjugated INPs and are approximately of 7–9 nm. The increase in size strongly indicates the binding of creatinine and urea onto INPs. In AFM images the INPs (Fig. 2) are of nearly spherical in size with 6 nm diameter and having tendency of agglomeration, whereas creatinine conjugated INPs (Fig. 3a, b) and urea conjugated INPs (Fig. 3c, d) are with nearly 9 nm diameter. It has been also observed that INPs are prone to quick agglomeration. But to the contrary the creatinine conjugated INPs and urea conjugated INPs are stable to coagulation because of its lack of free reactive surface.

### Interpretation of FT-IR spectra

**Fig. 4:**
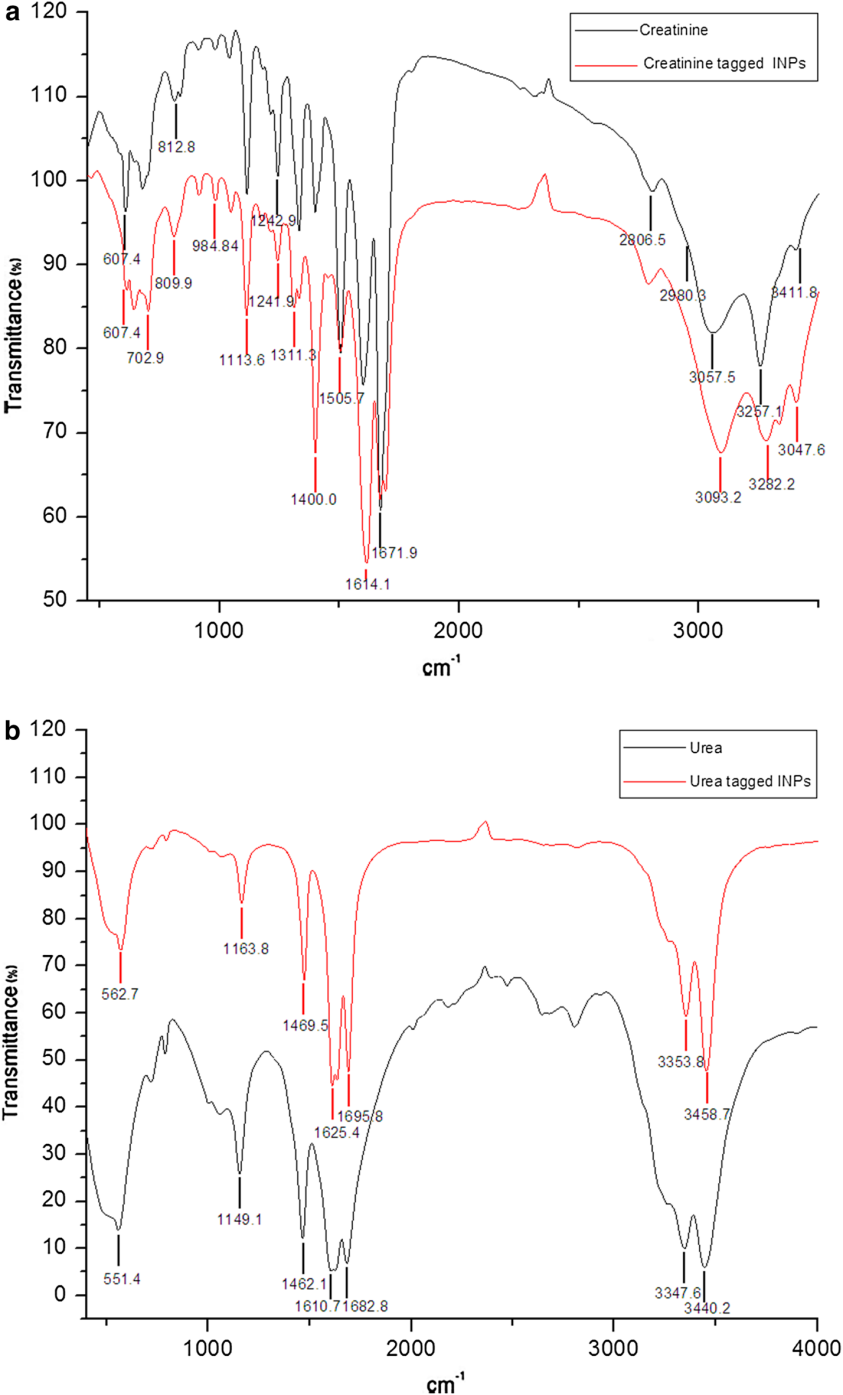
**a** FT-IR of creatinine (*black*) and creatinine tagged INPs (*red*) and **b** FT-IR of urea (*black*) and urea tagged INPs (*red*)

Interaction and binding of creatinine to the INPs were confirmed by taking FT-IR spectra of creatinine and creatinine tagged INPs. The FTIR spectrum of bare INPs does not show any characteristic peaks (Sharifi et al. 2013). Figure 4a shows FT-IR spectra of creatinine (black) and creatinine tagged INPs (red) in the region of 450–3500 cm^**−**1^. The fundamental modes of FT-IR in creatinine are the stretching and bending of N–H bond of the amino group present (Jerônimo et al. 2012). The creatinine molecule possesses one free NH_2_ group and hence one expects one symmetric and one asymmetric N–H stretching vibrations. In all the primary aromatic amines the N–H stretching frequency occurs in the region 3300–3500 cm^−1^. The FTIR spectrum of creatinine showed characteristic peaks at 3257 and 3057 cm^−1^, respectively due to one symmetric and one asymmetric N–H stretching vibrations (Jerônimo et al. 2012). Broad bands indicate the presence of the amine form in the structure. The observed low values of bands are due to the participation in the H-bonding interactions. The band observed at 1671 cm^−1^ is due to the C=O stretching mode (Pezzaniti et al. 2001). The NH_2_ out-of-plane deformation frequency is found in the 665–900 cm^−1^ region (NH_2_ wag) for primary amines. Here the bands observed at 841 and 812 cm^−1^ are as the fundamental due to the wagging modes of the amino group. The C–H stretching wave numbers of N bonded methyl group are lower than methyl groups attached to carbon atom. The *N*-methyl symmetric C–H stretch occurs from 2805 to 2780 cm^−1^ (Trendafilova et al. 1991). The bands at 2806 and 2980 cm^−1^ in the solid phase were assigned to the C–H symmetric and asymmetric stretching modes of N-CH_3_ group, respectively. Some measurable differences in the IR spectra of pure creatinine and creatinine tagged INPs were detected. When creatinine binds with INPs the N–H stretching vibration of NH_2_ group is much affected and found to shift to higher wavenumbers because when the aromatic ring nitrogen binds to metal, the ring stretching wave numbers shift to higher wave numbers. In the bound condition one symmetric and one asymmetric N–H stretching vibrations occur at 3282 and 3093 cm^−1^, indicating the presence less intermolecular hydrogen bonding. The observed bands corresponding to stretching vibrations γ←(NH_2_) and their wave numbers are found to be higher in value in case of creatinine tagged INPs than those of free creatinine and the −NH_2_ group of creatinine involved in interaction with the INPs. Thus for the creatinine-conjugated INPs the structural heterogeneity became less and the possibility of intermolecular hydrogen bonding among different conformers decreased. In the spectrum of urea (Fig. 4b) the asymmetric and symmetric N–H stretching bands can be found at 3440 and 3347 cm^−1^, respectively (Fischer and McDowell 1960). The band observed at 1682 cm^−1^ is due to the C=O stretching mode. The corresponding vibration wave numbers for urea tagged INPs analogues are found at 3458 and 3353 cm^−1^ (Jung et al. 2004). Solid urea showed characteristic but broad urea bands when it did not bind to the INPs, however most of the FT-IR bands were sharper in the urea tagged INPs.

### X-ray diffraction study

**Fig. 5:**
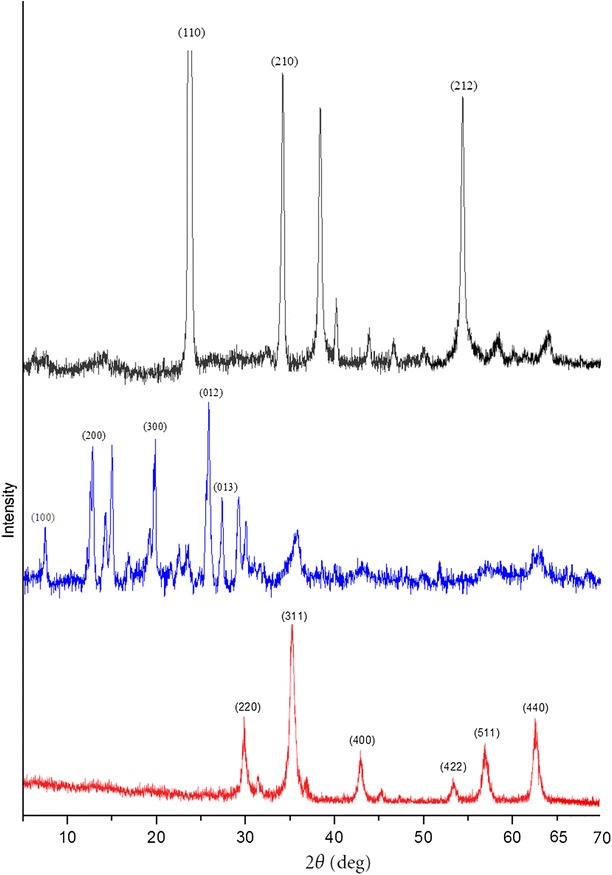
XRD patterns of urea tagged INPs (*black*), creatinine tagged INPs (*blue*) and INPs (*red*)

X-ray diffraction studies produce the long range order produced as a consequence of very short range interactions. Figure 5 shows the X-ray diffraction patterns of INPs, creatinine tagged INPs and urea tagged INPs.

For all the reflections correspond to INPs are very close to those of the standard data of Fe_3_O_4_ alone in powder diffraction PDF card (JCPDS No. 82n1533) (Banerji et al. 2012a). The presence of sharp and intense peaks confirmed the formation of highly crystalline INPs. The appearance of sample diffraction peaks at 2θ = 30.16°, 35.70°, 43.33°, 53.60°, 57.10°, and 62.9° corresponded to the (220), (311), (400), (422), (511) and (440) crystal planes of Fe_3_O_4_ respectively, which indicated that the resulting particles were Fe_3_O_4_, with structures of cubic crystal (Banerji et al. 2012). The X-ray diffraction pattern and the main diffraction angles of creatinine tagged INPs agreed with the data of creatinine. However, for creatinine tagged INPs the intensities of the diffraction peaks at 2θ = 7.4°, 19.9°, 25.8°, and 27.5° are due to the (100), (200), (300), (012) and (013) planes of creatinine molecule (Sakata et al. 2005). In case of urea tagged INPs the main diffraction angles agreed with the data of urea. However, the intensities of the diffraction peak at 2θ = 22.6°, 33.1° and 65.5° are corresponds to the (110), (210) and (212) planes of urea (Fernández-Bertrán et al. 2000). For creatinine tagged INPs and urea tagged INPs the indices corresponding to Fe_3_O_4_ are present. This reveals that the modification of INPs surface with creatinine or urea does not result in the phase change of Fe_3_O_4_.

### Application and future scopes

The INPs are nontoxic in nature to the human body which was earlier reported by our group (Banerji et al. 2012a). The binding of creatinine and urea into the INPs may be useful for the dialysis patients if this basic research is successfully translated and applied into dialysis technology. Dialysis is based on the principle of osmosis and the rate of osmosis may be enhanced by employing INPs externally into the dialyzer. It is proposed that the INPs may capture more creatinine and urea from the blood thereby shifting the equilibrium more towards right. Hence it is believed that urea and creatinine clearance may improve, this way by making the dialysis more efficient.

## Conclusion

All the analytical techniques (DLS, TEM, AFM, FT-IR, and Powder-XRD) have unambiguously proved the strong binding of creatinine and urea with INPs. In our laboratory, currently we are pursuing the dialysis study and will disclose the result in due time. In the present study we have successfully tagged creatinine and urea to the INPs. In this communication, the detailed binding studies are disclosed.
